# Supplementary material for: Biofouling Mitigation Approaches during Water Recovery from Fermented Broth via Forward Osmosis
Source: Membranes (Basel). 2020 Oct 27;10(11):307. doi: 10.3390/membranes10110307 (PMC7693741; doi:10.3390/membranes10110307)
Supplement: Supplementary file 1 [file membranes-10-00307-s001.pdf]

Supplementary material

Biofouling Mitigation Approaches during Water Recovery from Fermented Broth via Forward Osmosis

Stavros Kalafatakis [1], Agata Zarebska [2], Lene Lange [1,**], Claus Hélix-Nielsen [2], Ioannis V. Skiadas [1] and Hariklia N. Gavala [1,*]

[1] Technical University of Denmark (DTU), Department of Chemical and Biochemical Engineering, Søltofts Plads 229, 2800 Kgs. Lyngby, Denmark; stkalaf@gmail.com (S.K.); lene.lange2@gmail.com (L.L.); ivsk@kt.dtu.dk (I.V.S.)
[2] Technical University of Denmark (DTU), Department of Environmental Engineering, Miljøvej 113, 2800 Kgs. Lyngby, Denmark; AGZ@novozymes.com (A.Z.); clhe@env.dtu.dk (C.H.-N.)
* Correspondence: hnga@kt.dtu.dk and hari_gavala@yahoo.com
**New address: BioEconomy, Research and Advisory, Karensgade 5, Copenhagen, 2500 Valby, Denmark

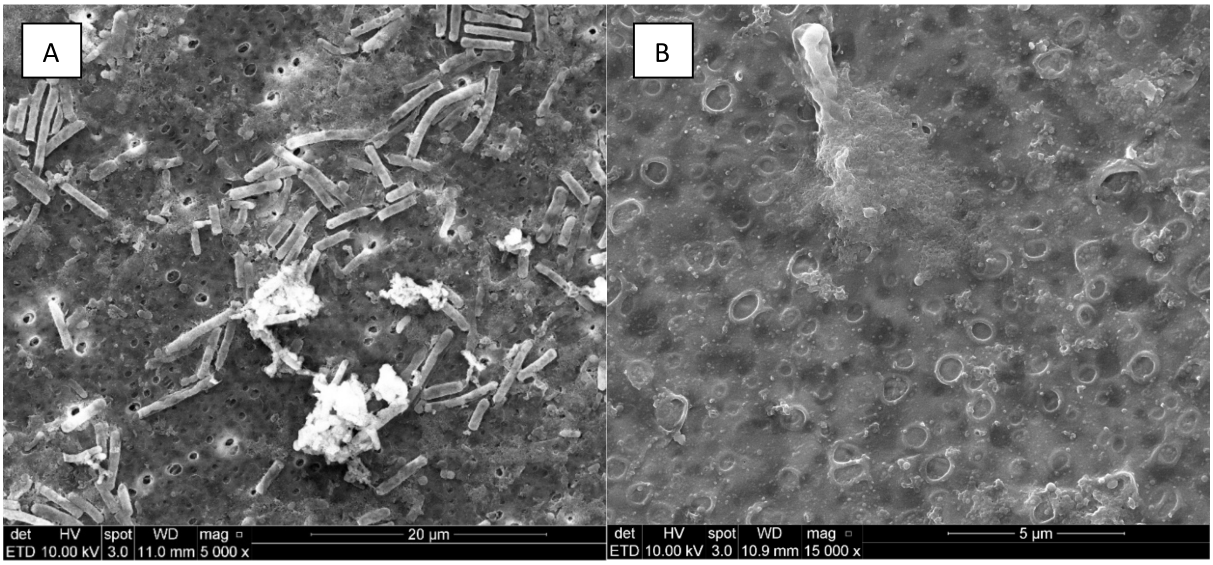

Figure S1. SEM image of active side of Aquaporin Inside^{TM} membrane surface (set up 4). A) 5000 magnification, B) 15000 magnification.

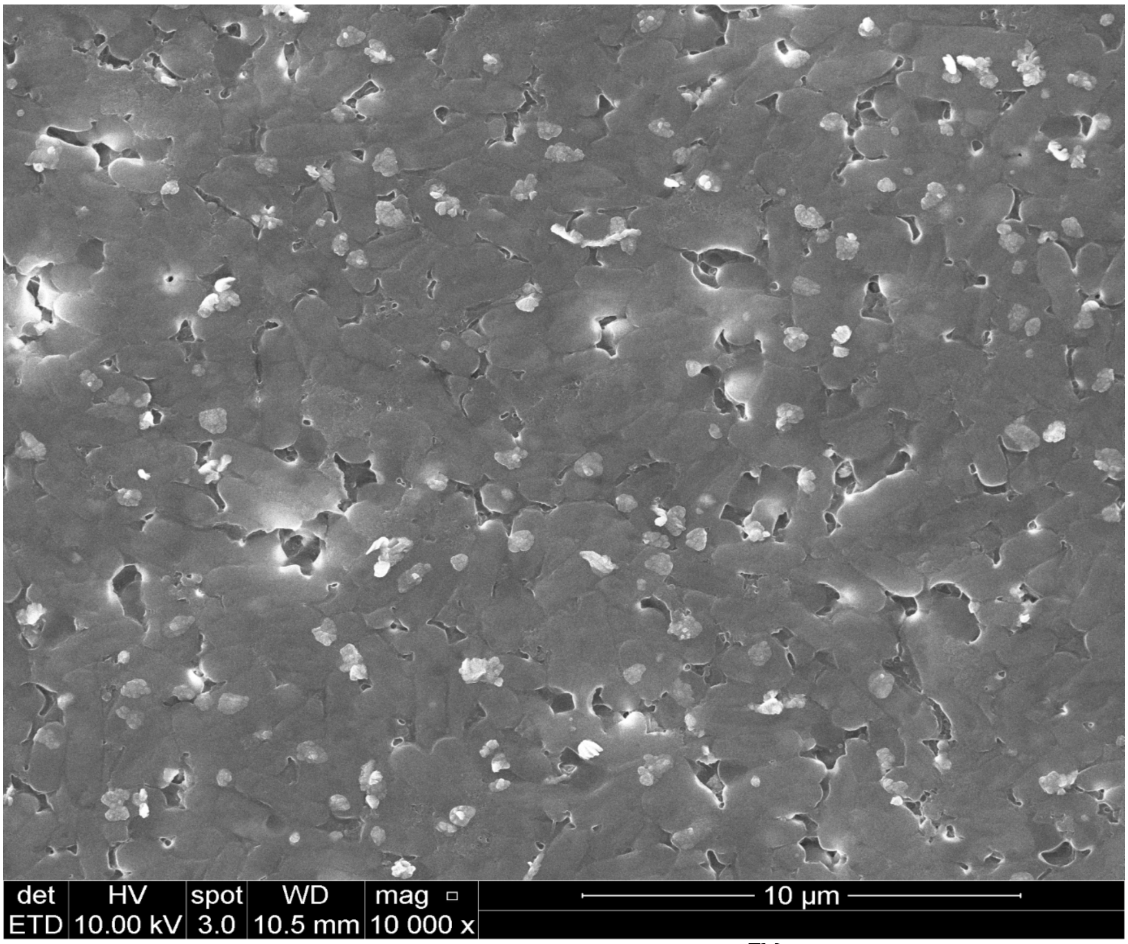

Figure S2. SEM image of active side of Aquaporin Inside™ membrane surface (set up 1).

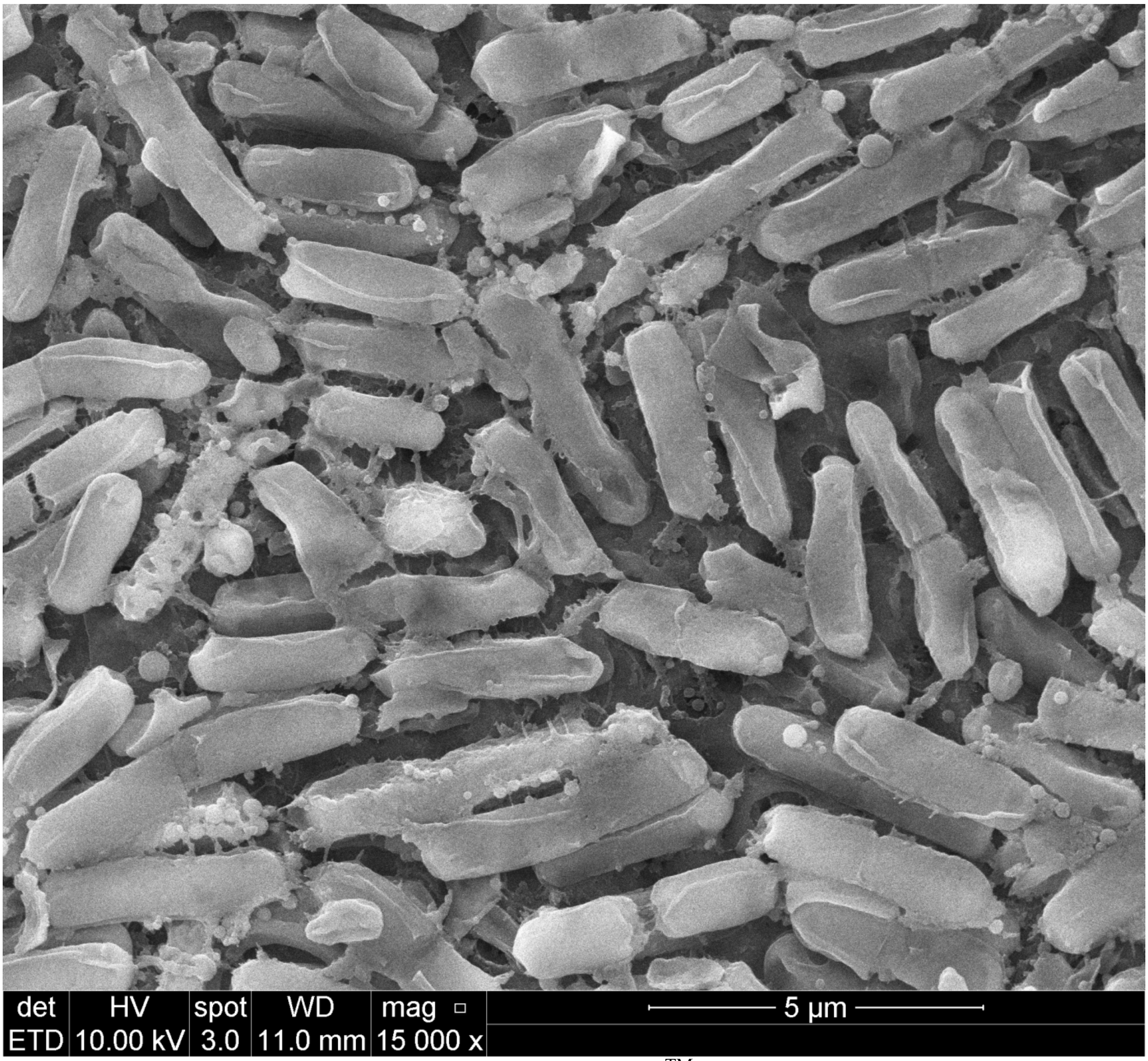

Figure S3. SEM image of active side of Aquaporin Inside^{TM} membrane surface (set up 2).

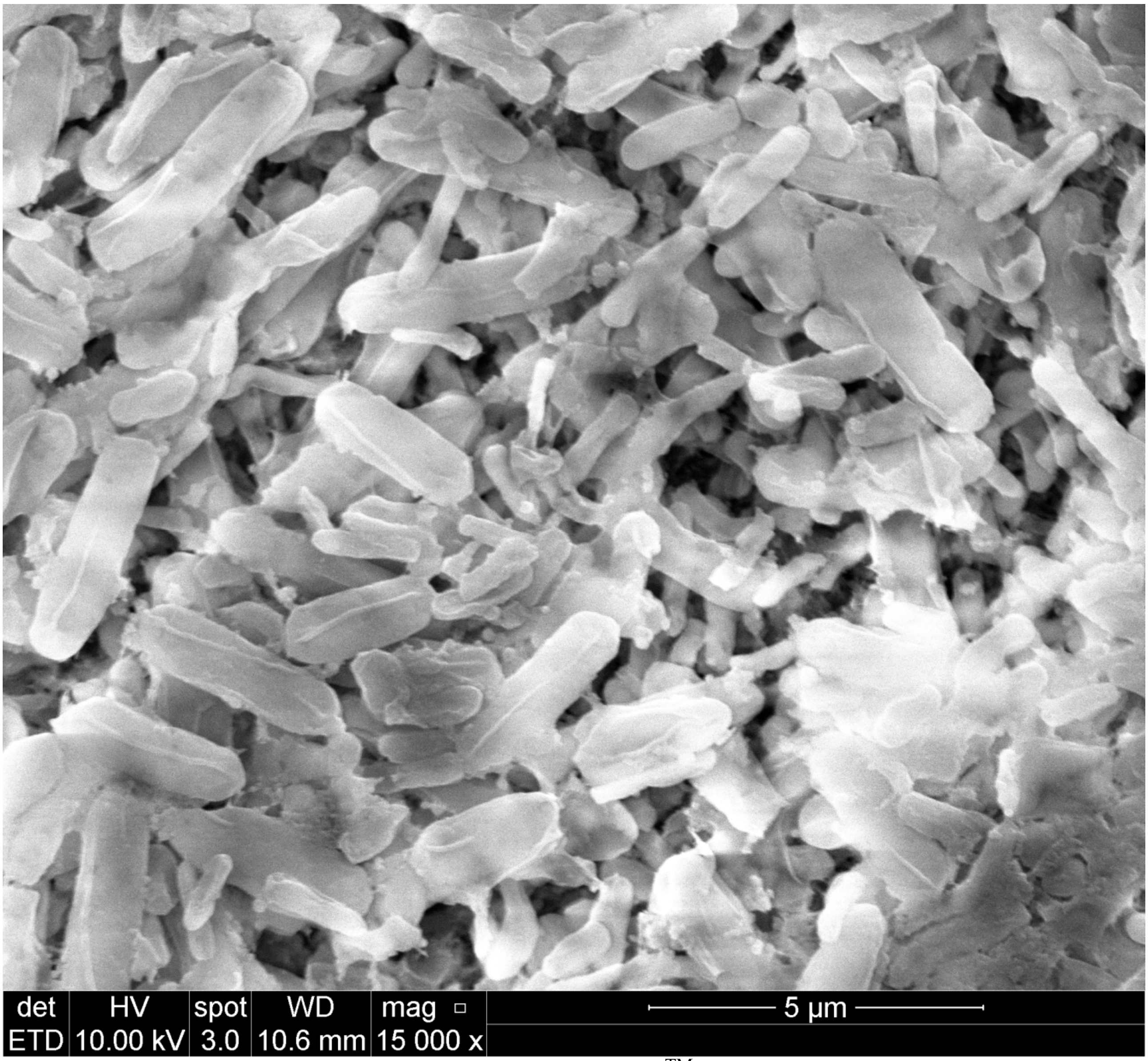

Figure S4. SEM image of active side of Aquaporin Inside^{TM} membrane surface (set up 3).

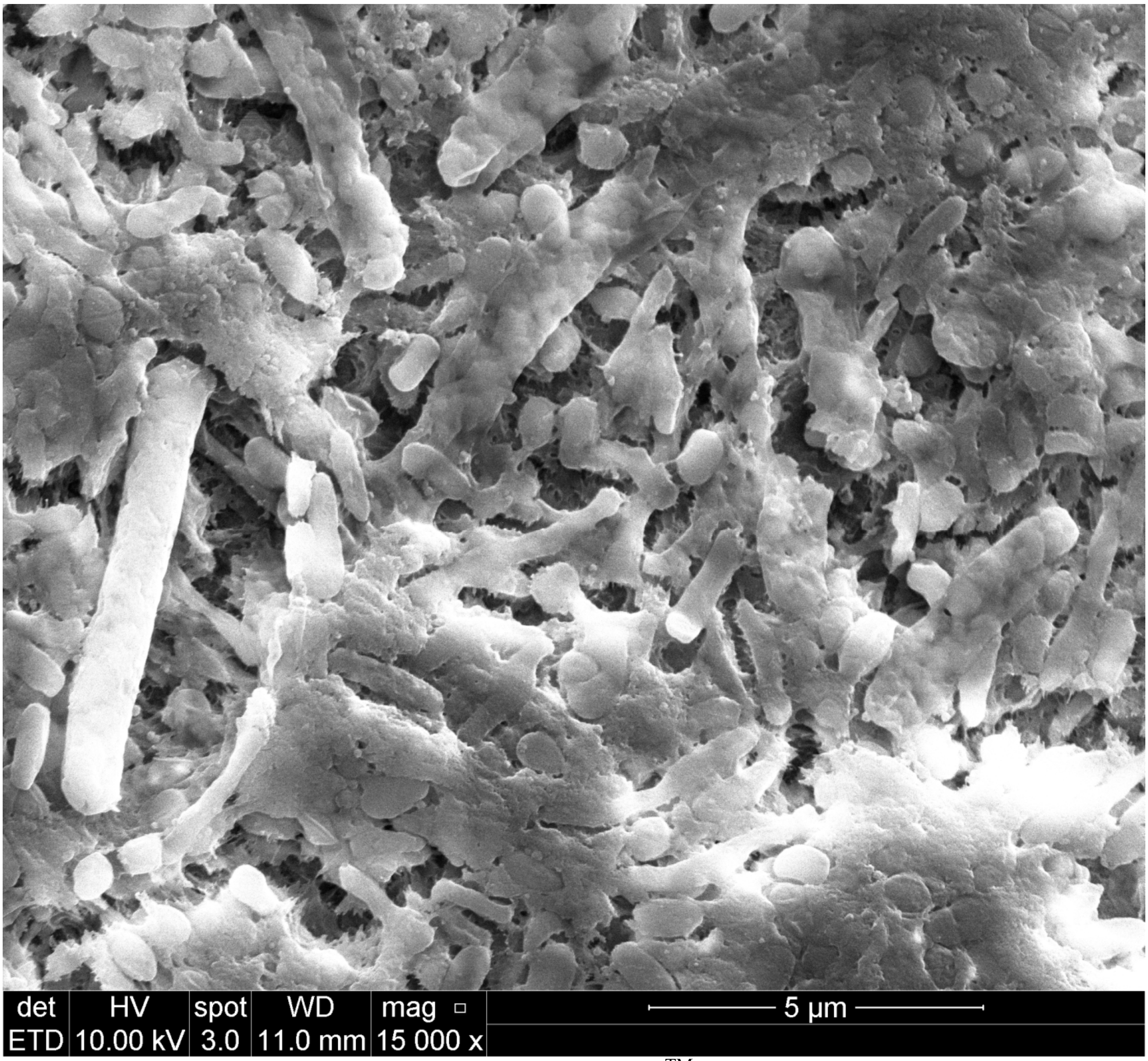

Figure S5. SEM image of active side of Aquaporin Inside^{TM} membrane surface (set up 5).

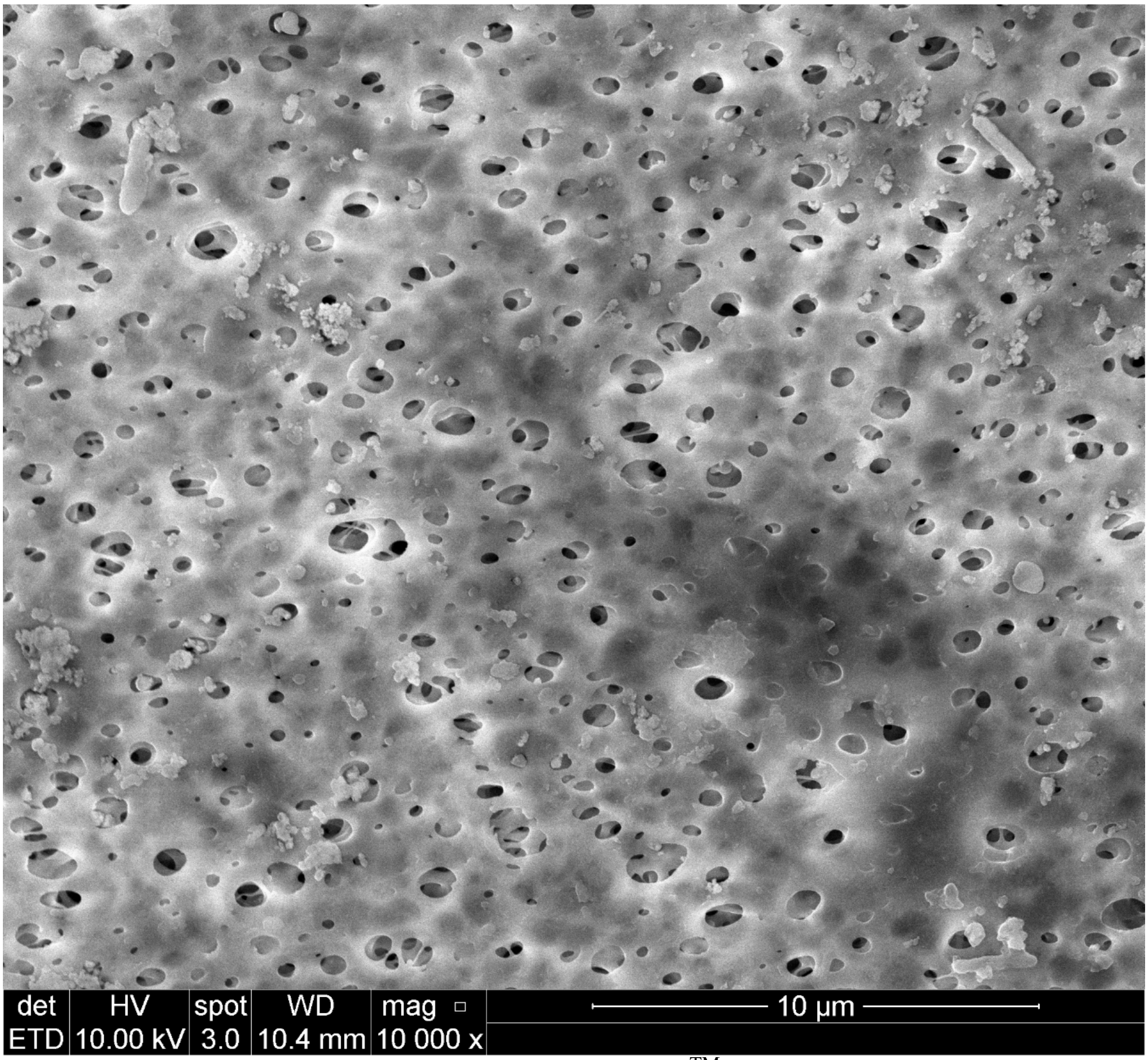

Figure S6. SEM image of support of Aquaporin Inside^{TM} membrane surface (set up 1).

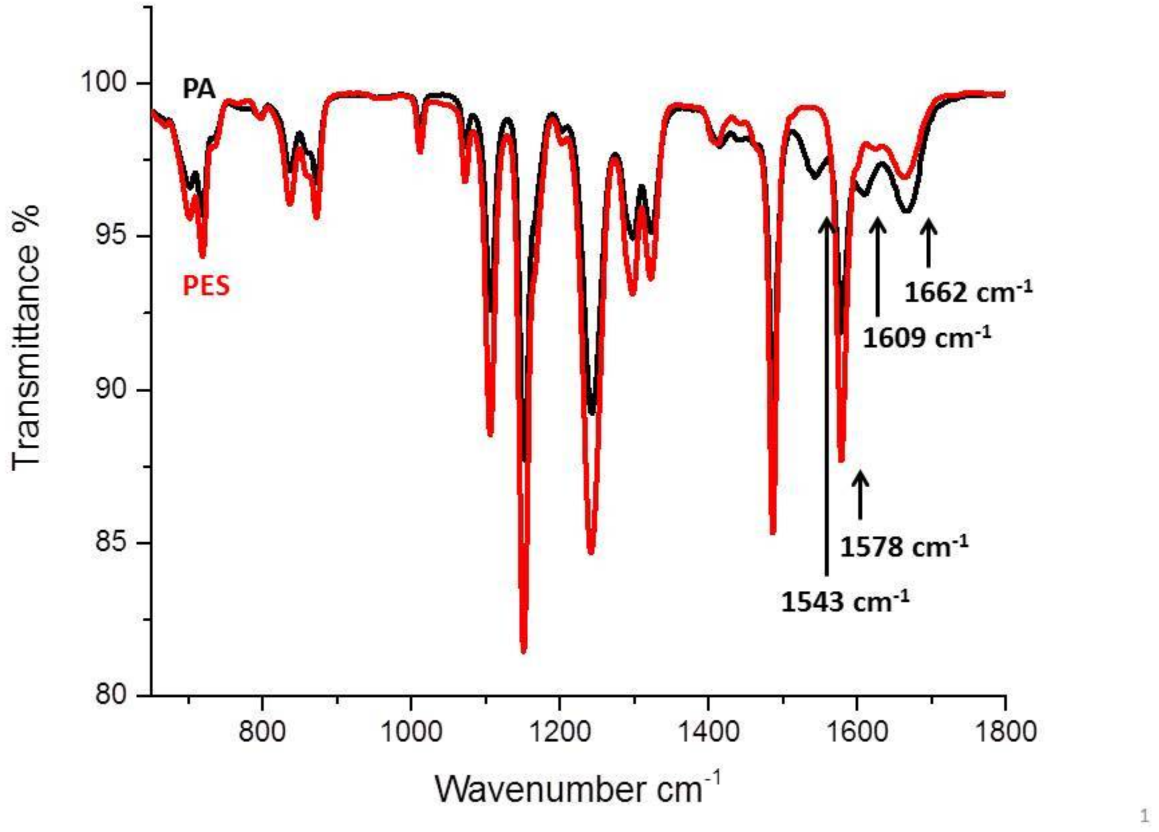

Figure S7. ATR-spectra of clean active polyamide (PA) and support polyethersulfone (PES) side of Aquaporin Inside^{TM} membrane.

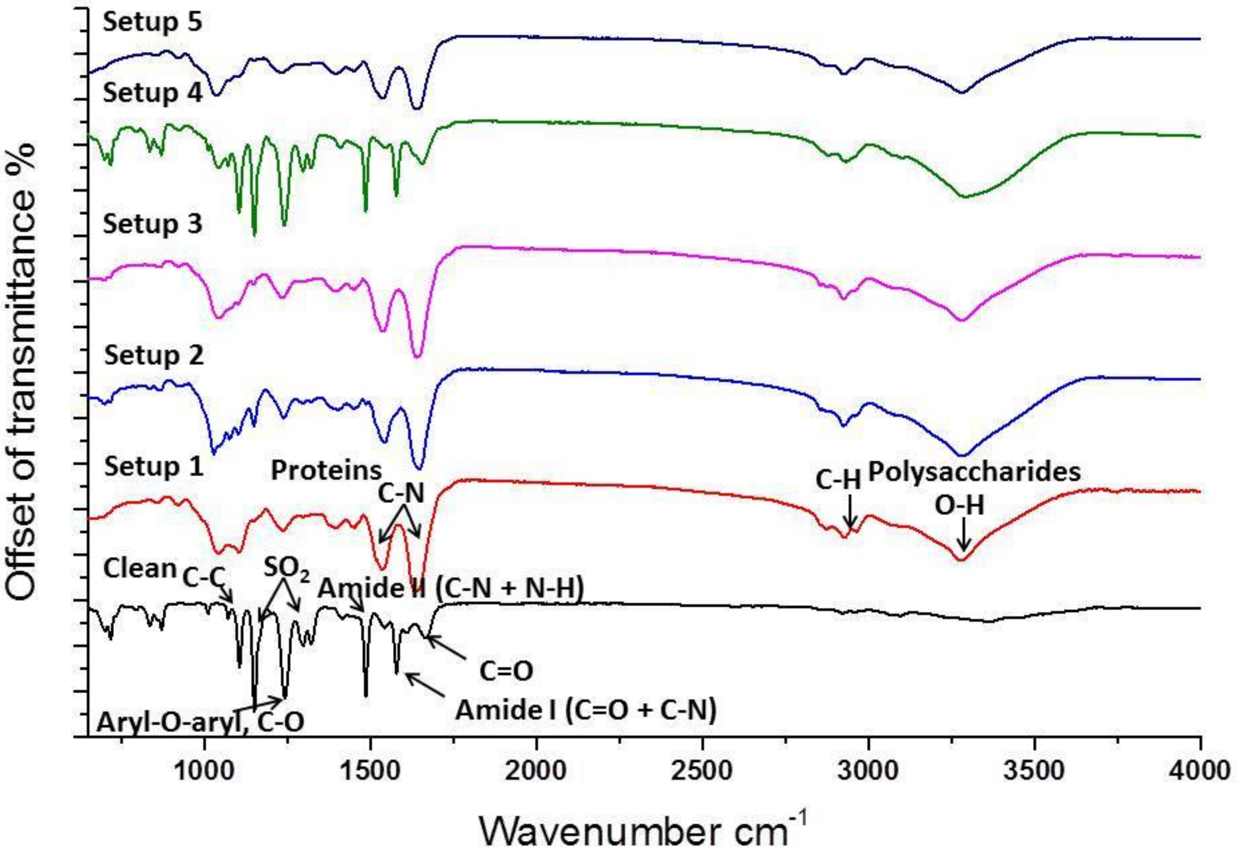

Figure S8. ATR-spectra of clean active side and fouled Aquaporin Inside^{TM} membranes.

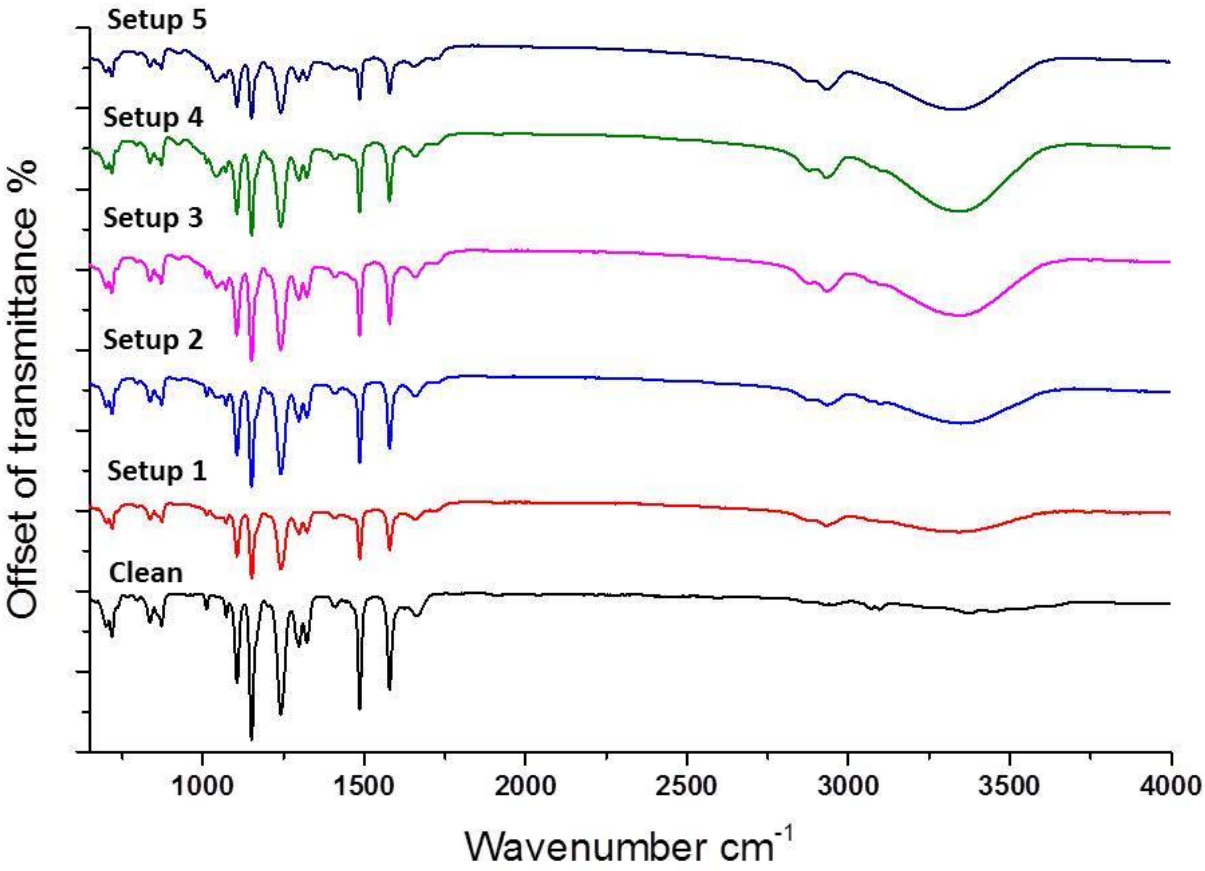

Figure S9. ATR-spectra of clean support side and fouled Aquaporin Inside™ membranes.
